# Harnessing Visible Light: Unraveling the Photocatalytic Water Splitting Activity of Ir–TiO_2_

**DOI:** 10.1021/acsaem.5c01776

**Published:** 2025-08-26

**Authors:** Moses D. Ashie, Chandra M. Adhikari, Gayani Pathiraja, Bishnu Prasad Bastakoti

**Affiliations:** † Department of Chemistry, 3616 North Carolina A&T State University, Greensboro, North Carolina 27411, United States; ‡ Department of Chemistry, Physics, and Materials Science, 3338 Fayetteville State University, Fayetteville, North Carolina 28301, United States; § Department of Nanoscience, Joint School of Nanoscience and Nanoengineering, 14616 University of North Carolina at Greensboro, 2907 East Gate City Blvd, Greensboro, North Carolina 27401, United States

**Keywords:** semiconductor, photocatalysis, water splitting, hydrogen evolution, TiO_2_

## Abstract

The quest to enhance the photocatalytic properties of TiO_2_ for hydrogen evolution in the visible region has necessitated its modification through various strategies. In this study, a one-pot solvothermally synthesized iridium-decorated titanium dioxide (Ir–TiO_2_) exhibits enhanced photochemical properties for splitting water in visible light. By varying the amount of Ir precursors, Ir-doped TiO_2_ and IrO_2_ composites with TiO_2_ were formed. Density functional theory (DFT) calculations reveal that Ir has localized d and f orbitals and that its oxide exhibits metallic character. When Ir replaces Ti as the dopant, energy levels appear near the Fermi level. At lower Ir concentrations, Ti still dominates, and Ti 3d hybridizes with Ir 5d, while O 2p interacts with Ir 5p, contributing to the narrowing of the band gap and modification of the chemical and electronic properties of TiO_2_. Photocatalytic hydrogen evolution experimental results revealed that Ir–TiO_2_ exhibits high activity with a yield of 1636.7 μmol h^–1^ g^–1^ compared to pristine (238.0 μmol h^–1^ g^–1^) and commercial (241.0 μmol h^–1^ g^–1^) TiO_2_. This can be attributed collectively to the reduction of the band gap for effective light absorption, a high surface area, and efficient charge transfer. The excellent recyclability and reusability of our materials demonstrate their long-term applicability as catalysts.

## Graphical abstract

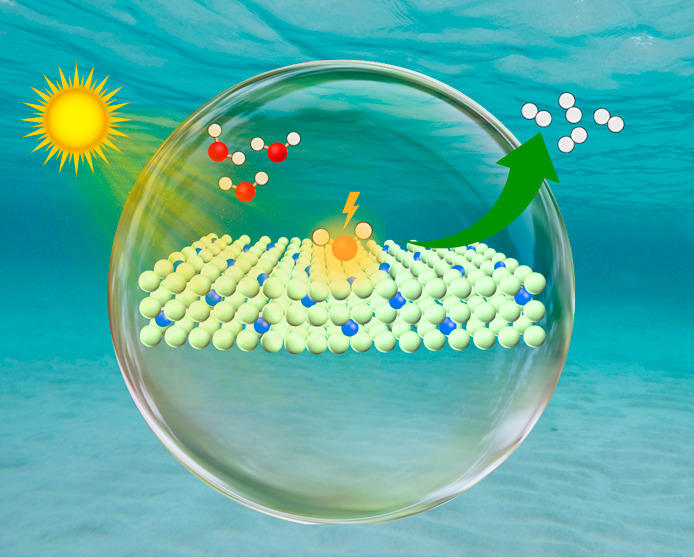

## 1 Introduction

The wide band gap of titanium dioxide limits its use as a photocatalyst for hydrogen generation in the visible region. This has been improved through surface modification to create defects, as well as coupling with other semiconductors and dopants.^1,2^ Coupling or composite formation with TiO_2_ helps to improve charge separation and visible light absorption.^3^ Doping helps narrow the band gap of TiO_2_, enabling light absorption in the visible region.^4^ TiO_2_ has reportedly been doped with metals and nonmetals, which helps create new energy levels below the conduction band (CB) or above the valence band (VB), reducing the band gap.^5^ Noble metals such as Ru, Au, Pt, Pd, and Ir have been studied to exhibit unique and superior properties in light absorption and other tunable properties of photocatalysts, which extend the photocatalytic response of TiO_2_ into the visible region, resulting in the enhancement of overall photocatalytic activity.^6,7^ Iridium has been extensively reported in complexes, metal–organic frameworks (MOFs), and other organic–inorganic composites for hydrogen generation and other photocatalytic applications, yielding varying results and providing insight for future material design.^8^ MOFs modified with an iridium complex were found to exhibit enhanced light absorption properties and maintain photostability. The fabricated material containing 0.2% of Ir yielded 446.4 μmol g^–1^ of hydrogen gas.^8^ Wang et al. also reported the use of iridium-based catalysts in the form of alloys, pyrochlore, perovskite, heteroelemental doping, core–shell structure, and supported catalysts, primarily for the oxygen evolution reaction.^9,10^ However, only a few studies have been performed to investigate photocatalytic hydrogen evolution using titanium dioxide-based iridium. Due to iridium’s exceptional catalytic properties, Ir–TiO_2_-based composites are expected to perform potentially better when used as photocatalysts. Yu et al. fabricated hollow TiO_2_ spheres and deposited amorphous iridium oxide nanosheets, which showed improved activity in acidic electrolytic water splitting.^11^ Iridium (1.35 wt %) atomically deposited on TiO_2_ nanosheets demonstrated low overpotential (41 mV at 10 mA cm^–2^) and a small Tafel slope of 42 mV dec^–1^, which are favorable for the hydrogen evolution reaction.^12^ Trang et al. used the photocatalytic water splitting technique to investigate the hydrogen evolution performance of the anatase–rutile–Ir composite, which yielded 48 and 23.5 μmol h^–1^ g^–1^ at 365 and 420 nm irradiation wavelengths, respectively.^13^

The electronic structure of iridium oxide electrodes active in water splitting revealed electronic defects, which were attributed to the improved performance of amorphous IrO_2_. In the OER investigation, Ngo et al. observed that the amorphous IrO_2_ and crystalline IrO_2_ composites exhibited a smaller Tafel slope compared to the separate amorphous and crystalline materials, indicating faster reaction kinetics. Based on these results, the amorphous/crystalline material was confirmed to achieve higher current densities at lower overpotentials.^14^ IrO_2_ material in its amorphous form, combined with a crystalline composite, has been studied to perform better than the crystalline IrO_2_.^15^

In this work, we investigated the effect of iridium on enhancing the photocatalytic ability of TiO_2_ in the solar light region. Using a one-step solvothermal technique, we fabricated iridium-doped TiO_2_ nanoparticles with good photocatalytic properties. By increasing the amount of the iridium component, the IrO_2_/TiO_2_ composite was formed, which demonstrated enhancements in the structural and photocatalytic properties of titanium dioxide. By varying the dopant concentration, we could identify the material with the best photocatalytic properties for the effective activity. The application of the synthesized material in the hydrogen evolution experiment demonstrated that our fabricated photocatalyst is highly efficient in photocatalytic water splitting for hydrogen evolution under solar light irradiation. Density functional theory (DFT) studies provided further insight into understanding the electronic properties of our material and the contribution of Ir and IrO_2_ in achieving highly improved photocatalytic properties for hydrogen evolution under visible light.

## 2 Experimental Section

### 2.1 Materials

Isopropyl alcohol (IPA), 99.9%, was from LabChem, USA. *N*,*N*-Dimethylformamide (DMF), 99.5%, was obtained from Fisher Chemical. Titanium (IV) isopropoxide, Ti­[OCH­(CH_3_)_2_]_4_ (TTIP), 97%, was from Alfa Aesar, USA. *N*-Methyl-2-pyrrolidone (NMP), 99.5%, was from Alfa Aesar, USA. Hydrogen hexachloroiridate (IV) hydrate, 99.9%, trace metal basis, was purchased from Sigma-Aldrich, USA. Poly­(vinylidene fluoride) (PVDF) was from Sigma-Aldrich, USA, with an average molecular weight of 534,000. TiO_2_ (Aeroxide, P25), 95%, from Sigma-Aldrich, USA, was used as a control sample. No further purification was performed on the reagents before usage.

### 2.2 Synthesis of Ir–TiO_2_ Samples

A uniform mixture of 60 mL of IPA and 20 mL of DMF was used as a solvent, and 0.5 g of Pluronic F-127 was dissolved in it. A 2 mL portion of titanium­(IV) isopropoxide was added to the resulting solution and stirred for 1 h. A known amount of hydrogen hexachloroiridate (IV) hydrate was added, stirred for 30 min, and then thermally treated at 180 °C for 20 h in a vacuum oven (VT6025), Thermo Scientific, Germany. The samples were dried in an oven at 60 °C after washing with ethanol and then annealed at 500 °C for 2 h at a rate of 3 °C/min using an OTF-1200X tubular furnace purchased from MTI Corporation, USA. Ir–TiO_2_-A, Ir–TiO_2_-B, and Ir–TiO_2_-C were used to denote samples containing 0.0069, 0.034, and 0.069 g of hydrogen hexachloroiridate (IV) hydrate, respectively. A pristine TiO_2_ sample synthesized without an Ir precursor was denoted as TiO_2_–P.

### 2.3 Characterization

An X-ray diffractometer (XRD) Rigaku Miniflex 600 model, USA, was used to obtain XRD spectra from 10° to 90° using CuK radiation (λ = 1.5417 Å), a current of 15 mA, an operational voltage of 40 kV, and a scan rate of 2°/min and operating under ambient conditions with a step size of 0.02°. The Debye–Scherrer equation was used to calculate the particles’ crystallite size. An X-ray photoelectron spectrometer Escalab Xi+ from Thermo Fisher, USA, aided in the analysis of the elemental and chemical composition of the samples. A field emission scanning electron microscope (Model JSM-IT800), Japan, and a transmission electron microscope (Model JEOL JEM-2100plus), Japan, coupled with electron dispersive X-ray spectroscopy (EDX), were used to study the morphology. The ImageJ software was used to calculate the interplanar distances and particle sizes. The vibrational response of the different particle compositions was studied using Fourier transform infrared spectrometry (FTIR, A217060), Shimadzu Corp., Japan, and Raman confocal microscopy (Horiba), USA, techniques. A Shimadzu IRTracer-100 spectrophotometer equipped with a modernized DLATGS detector was used to measure FTIR spectra from 400 to 4000 cm^–1^. A DiffusIR reflectance accessory (PIKE Technologies) was integrated into a UV–vis spectrometer (Evolution Pro, EV3Z315002, Thermo Scientific), USA, to facilitate recording of the light response of the samples. Brunauer–Emmett–Teller (BET) surface analysis was performed with a BET analyzer (Micromeritics ASAP 2060), USA. Density functional theory (DFT) results were obtained from first-principles ab initio quantum mechanical calculations.

### 2.4 Electrochemical Characterization

A 100 μL portion of a 1% w/v PVDF in NMP solution was used to disperse 4 mg of finely powdered sample, aided by sonication for 1 h. The resulting suspension was coated onto a copper (Cu) substrate by drop casting and repeated drying, forming an electrode of a 1 cm^2^ area. The prepared electrode was then dried in an oven at 60 °C for 12 h, and the electrochemical response was verified via an electrochemical workstation, 700E series, CHI Instruments, USA. Electrochemical impedance spectroscopy (EIS), cyclic voltammetry, amperometry technique for transient current switched on-and-off test, and stability of our samples were examined via a three-electrode system with the Cu substrate serving as the working electrode, a platinum electrode serving as the counter electrode, and an Ag/AgCl electrode serving as a reference electrode. A 0.5 M Na_2_SO_4_ solution was used as an electrolyte.

### 2.5 Photocatalytic Testing

The photocatalytic hydrogen evolution was performed in a double-walled photoreactor using solar light irradiation of AM 1.5G from a solar simulator (model PEC-L01, China, with an intensity of 200 mW cm^–2^ set). The Xe lamp of the solar simulator emits light across a range of approximately 350–1100 nm. A 30 mg portion of the powder material was added to 50 mL of a 10% methanol solution in deionized water, serving as the reaction medium with a stirring rate of 250 rpm. The reactor system was purged for 5 min with nitrogen and irradiated with light for 3 h. An infrared temperature detector (Custom IR-300), Japan, was used to monitor the temperature within the reactor, and the light intensity was measured using a light meter LT300 (Extech Instruments), USA. The hydrogen gas was quantified using a gas chromatograph (model 8610C from SRI), USA, with argon as the carrier gas and a thermal conductivity detector. The gas chromatograph was calibrated with standard H_2_ (100 ppm) calibration gas from ARC3 Gases, USA.

### 2.6 Computational Methods

First-principles ab initio quantum mechanical calculations were performed using the Vienna Ab Initio Simulation Package.^16,17^ The projector augmented wave method was used to account for/describe ion–electron interactions, while the exchange–correlation potentials were approximated by the Perdew–Burke–Ernzerhof functional.^18,19^ Atomic coordinates were fully relaxed to optimize the crystal structures to their minimum energy and force, with energy and force convergence criteria of 10^–6^ eV and 10^–2^ eV/Å, respectively. On-site Coulomb repulsion between electrons in a band was appropriately addressed using Hubbard U parameters, namely, 6.5 eV for Ti and 2.5 eV for Ir. A 520 eV plane-wave kinetic energy cutoff was used. The Γ-centered *k*-point meshes of 12 × 12 × 12 and 2 × 2 × 4 were used for Brillouin zone sampling for a single unit cell of pristine TiO_2_ and iridium (Ir)-doped TiO_2_ supercell, respectively. The initial crystal structure of TiO_2_ was adopted from the Materials Project.^20^

## 3 Results and Discussion

The iridium-decorated TiO_2_ microspheres were synthesized by using the solvothermal method. The Pluronic F127 surfactant is incorporated in this synthesis to contribute as a structure-directing agent through micelle formation. The self-assembly of TTIP^21^ was accelerated upon the addition of Ir precursors (Figure S1). The synergic contribution of the surfactant and mixed solvents leads to the formation of Ir-loaded TiO_2_. The polymer was removed after annealing at 500 °C. The removal was confirmed with FTIR (Figure S2). The annealing not only eliminates the polymer but also induces crystallinity.^22^ The distinct peaks from Ir/IrO_2_ were not observed in XRD at 500 °C. However, IrO_2_ peaks were observed in the Ir–TiO_2_ sample above 500 °C (Figure S3). This agrees with the literature that IrO_2_ crystals were well formed at temperatures above 500 °C.^23^ The higher temperature (above 500 °C) required for effective crystallization of the IrO_2_ can be attributed to surface passivation (Figure S4) by the surfactant.^24^ In comparison, IrO_2_ XRD peaks were observed at the 500 °C annealing temperature for Ir–TiO_2_ synthesized without surfactants (Figure S5). It can be concluded that at 500 °C, residual carbonaceous species^25,26^ from the surfactant suppressed the crystallinity to form amorphous IrO_2_ while the TiO_2_, remained highly crystalline. As shown in Figure S3, crystallinity was enhanced in the surfactant-assisted Ir–TiO_2_-B, and Ir–TiO_2_-C samples at a calcination temperature of 600 °C. The amorphous state of the IrO_2_ is evident since a study by Musić et al., using TGA and XRD, reported the formation of crystals of IrO_2_ at about 460 °C.^27^ XRD measurement (Figure 1a) further revealed the crystallinity of the synthesized materials with TiO_2_ exhibiting the anatase phase (standard PDF no. 00-021-1272). At a calcination temperature of 600 °C, IrO_2_ peaks were observed at 28.11°, 34.75°, and 53.96°, corresponding to 110, 101, and 211 crystal planes (Figure S3), respectively, with standard PDF no. 00-015-0870 for the surfactant-assisted sample. At 500 °C, surface elemental analysis by X-ray photoelectron spectrometry (XPS) revealed average Ir atomic percent values of 0.11%, 0.73%, and 0.93% for Ir–TiO_2_-A, Ir–TiO_2_-B, and Ir–TiO_2_-C samples, respectively.

**Figure 1:**
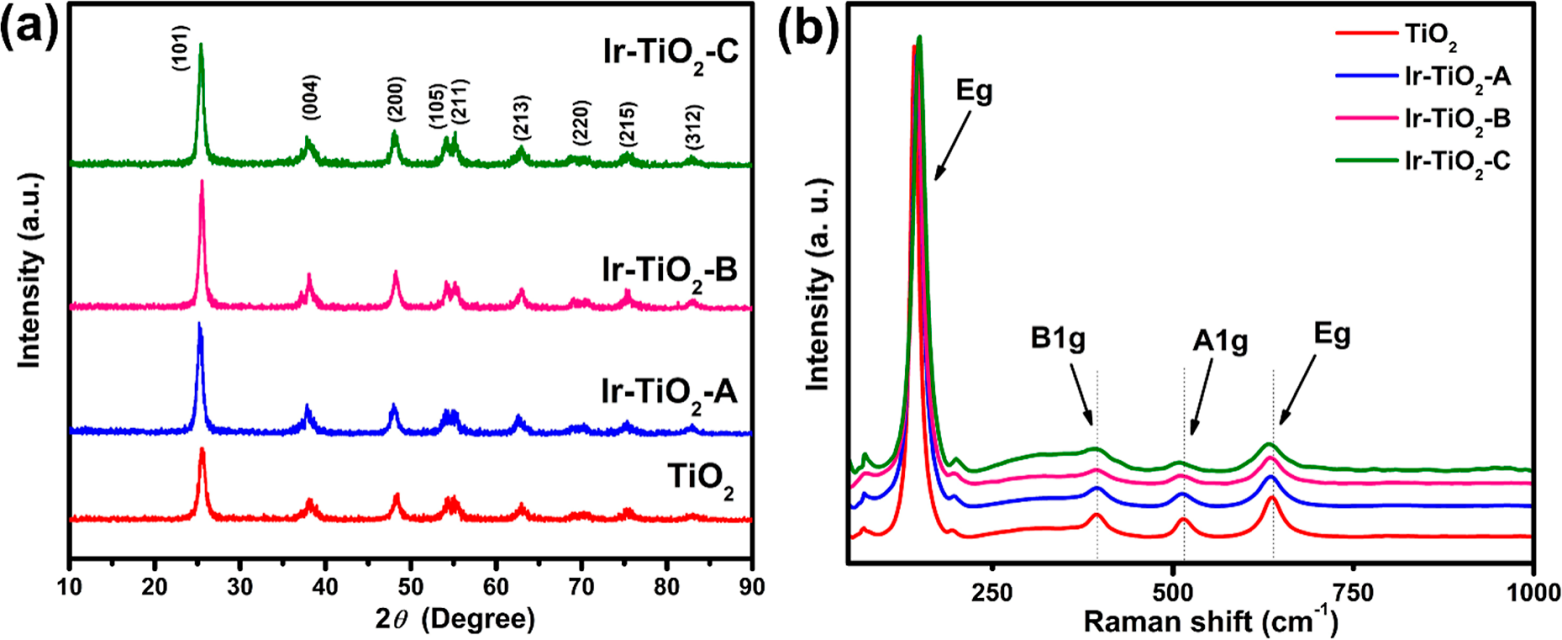
(a) XRD patterns and (b) Raman shift of pristine TiO_2_ and synthesized Ir–TiO_2_ samples.

All synthesized samples exhibited a blue shift at 395.8 cm^–1^ (B_1g_), 516.0 cm^–1^ (A_1g_), and 638.9 cm^–1^ (E_g_) to shorter wavelengths in the Raman analysis with the Ir–TiO_2_–C experiencing a more significant shift compared to the Ir–TiO_2_-A and Ir–TiO_2_-B samples (Figure 1b). A red shift was observed at the 141.4 cm^–1^ (E_g_) vibrational band for all samples. The Raman analysis was carried out at an excitation wavelength of 785 nm. The shift in the peaks confirms the doping effect on TiO_2_ after incorporating Ir during synthesis. Broadening of peaks was observed in the order Ir–TiO_2_-C > Ir–TiO_2_-B > Ir–TiO_2_-A. Characteristic Raman bands typical for iridium oxide at about 561 cm^–1^ (E_g_), 723 cm^–1^ (B_2g_), and 752 cm^–1^ (A_1g_) were absent.^28^ This could be attributed to the amorphous nature of IrO_2_ in the composite. The variation in peak intensities, widths, and peak shift indicates modification of the TiO_2_ lattice due to Ir dopants. BET analysis results (Table S1) show an increase in the surface area of Ir–TiO_2_-C (112.0 m^2^/g) compared to TiO_2_-only (106.9 m^2^/g) and the other samples.

High-resolution transmission electron microscopy (HR-TEM) operated at an accelerating voltage of 200 kV from JEOL 2100PLUS with STEM/EDS capability was used to examine the morphology, crystallinity, and elemental distribution of the Ir–TiO_2_-C sample. The TEM image (Figure 2a) demonstrates the morphology of the microstructures of the Ir–TiO_2_-C sample. The higher magnification of the highlighted area reveals the lattice spacings of this novel material (Figure 2b), and the measured value of 0.34 nm aligns with the high intensity of the (101) crystal plane in Figure 2c, which was also observed in the XRD analysis and corresponds to TiO_2_. The polycrystalline nature of microstructures is further confirmed by their selected area electron diffraction (SAED) pattern (Figure 2d), which consists of a characteristic ring pattern with many brighter spots from their individual crystals. The angular-dark field-scanning transmission electron microscopy (ADF-STEM) image in Figure 2e, with EDX elemental mapping in Figure 2f–i, demonstrated the homogeneous elemental distribution of Ti, O, and Ir in these microspheres.

**Figure 2:**
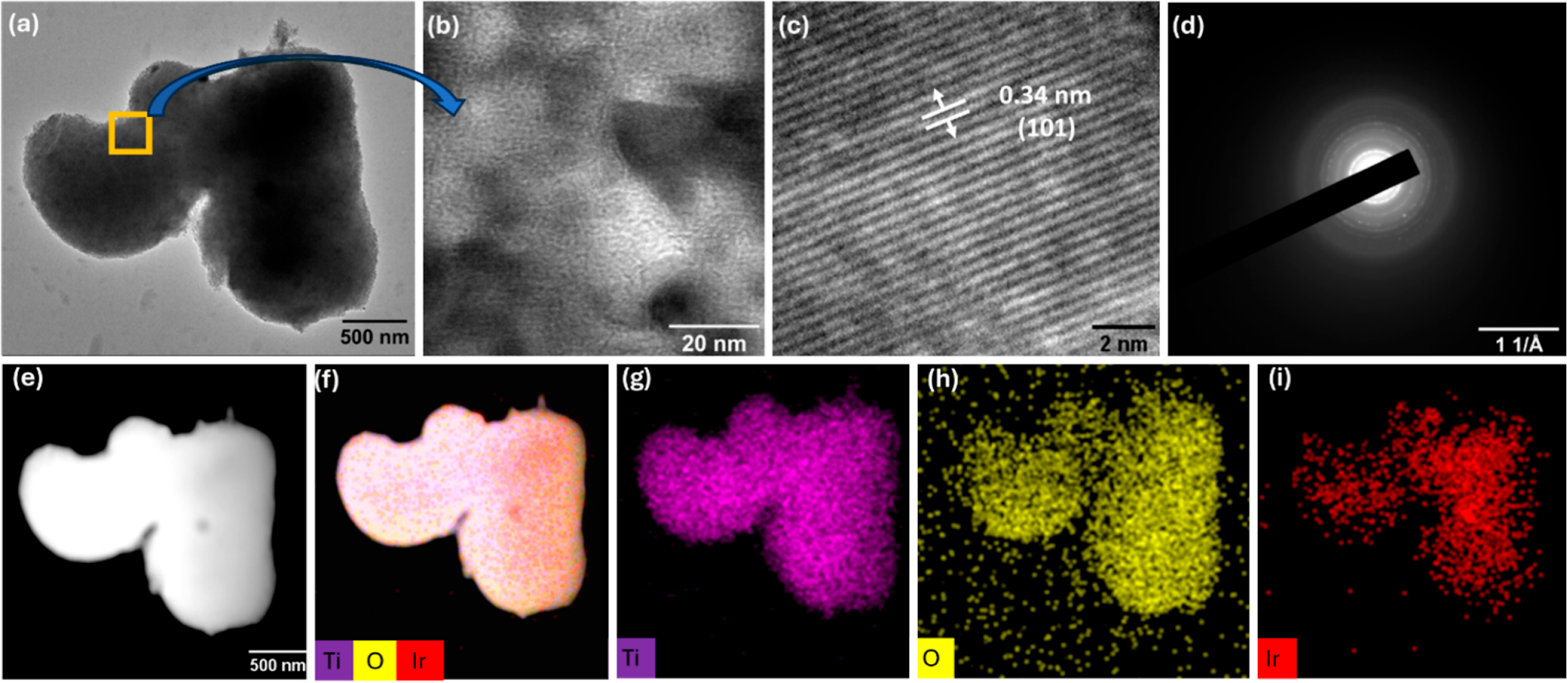
(a) TEM image of Ir–TiO_2_-C microspheres. (b) HR-TEM image at 300 kx that magnifies the region of the orange rectangular square; (c) its lattice spacing of the (101) crystal plane; (d) its SAED pattern; (e) ADF-STEM image of microspheres; and (f) overlay mapping and (g–i) individual elemental mapping of Ti, O, and Ir, respectively.

The elemental composition and chemical states of the samples were analyzed by using XPS, and the presence of Ir, Ti, and O was revealed in the survey spectrum (Figure S6). Two intense peaks in the deconvoluted Ti 2p peaks at 459.2 and 464.8 eV correspond to Ti 2p_3/2_ and Ti 2p_1/2_, respectively, indicating the presence of Ti^4+^ in the amorphous IrO_2_/crystalline TiO_2_ composite.^29^ From the XPS elemental scan, the doublets that correspond to Ir 4f_7/2_ and Ir 4f_5/2_ were seen after deconvolution. The Ir 4f_7/2_ and Ir 4f_5/2_ binding energies (BE) of 63.9 and 66.1 eV can be attributed to Ir in the 4+ oxidation state. These BEs are close to reported results^15^ with a slight shift toward higher BE. The observations can be attributed to the strong interaction between the IrO_2_ particles and the TiO_2_ crystals, as well as the amorphous nature of the IrO_2_ in the composite. The absence of peaks at about 60 eV confirms the absence of Ir metals^30^ and nondoping in sample Ir–TiO_2_–C, with the corresponding absence of peaks in XRD. The deconvoluted O 1s peak from oxygen showed a peak at 530.1 eV, typical of metal-oxide bonding (Ti–O). 530.1 eV and other BE at 530.4 eV can be attributed to a metal–oxygen bonding (Ti–O) in a different chemical environment.^31^ The peak at 531.2 eV is indicative of surface hydroxyl (−OH) groups due to surface-adsorbed water.^32^ The additional hydrogen bond increased the BE of the O 1s compared to that of the O bonded to the metal.

Figure 3a–d shows the effect of the composite on the Fermi level and band alignments in our materials. No carbon correction of our data was performed, as the position of the adventitious carbon peak (284.9 eV) was nearly identical to the binding energy of the C 1s peak (typically around 284.6 eV). XPS-VB analysis revealed differences in the valence band maximum (VBM) of all samples after incorporation of Ir, indicating an interaction between iridium and TiO_2_. Differences in BE between the O 2s and O 2p states were also observed after doping. All spectra exhibited broad peaks at a BE of about 5 eV, which can be attributed to electrons from the valence band states of O 2p and Ti 3d orbital hybridization.^33,34^ Changes observed in the Fermi-level position are due to the doping effect.^35^ The increase in the VBM of all doped samples, Ir–TiO_2_-A (1.46 eV) (Figure 3b), Ir–TiO_2_-B (1.42 eV) (Figure 3c), and Ir–TiO_2_-C (1.45 eV) (Figure 3d), compared to TiO_2_ (1.05 eV) (Figure 3a), with a corresponding shift in CBM values of Ir–TiO_2_-A (−1.35 eV), Ir–TiO_2_-B (−1.45 eV), and Ir–TiO_2_-C (−1.46 eV) compared to TiO_2_ (−2.07 eV), is an indication of significant narrowing of the band gap. The Fermi level shifts closer to the conduction band, indicating the formation of an n-type material.

**Figure 3:**
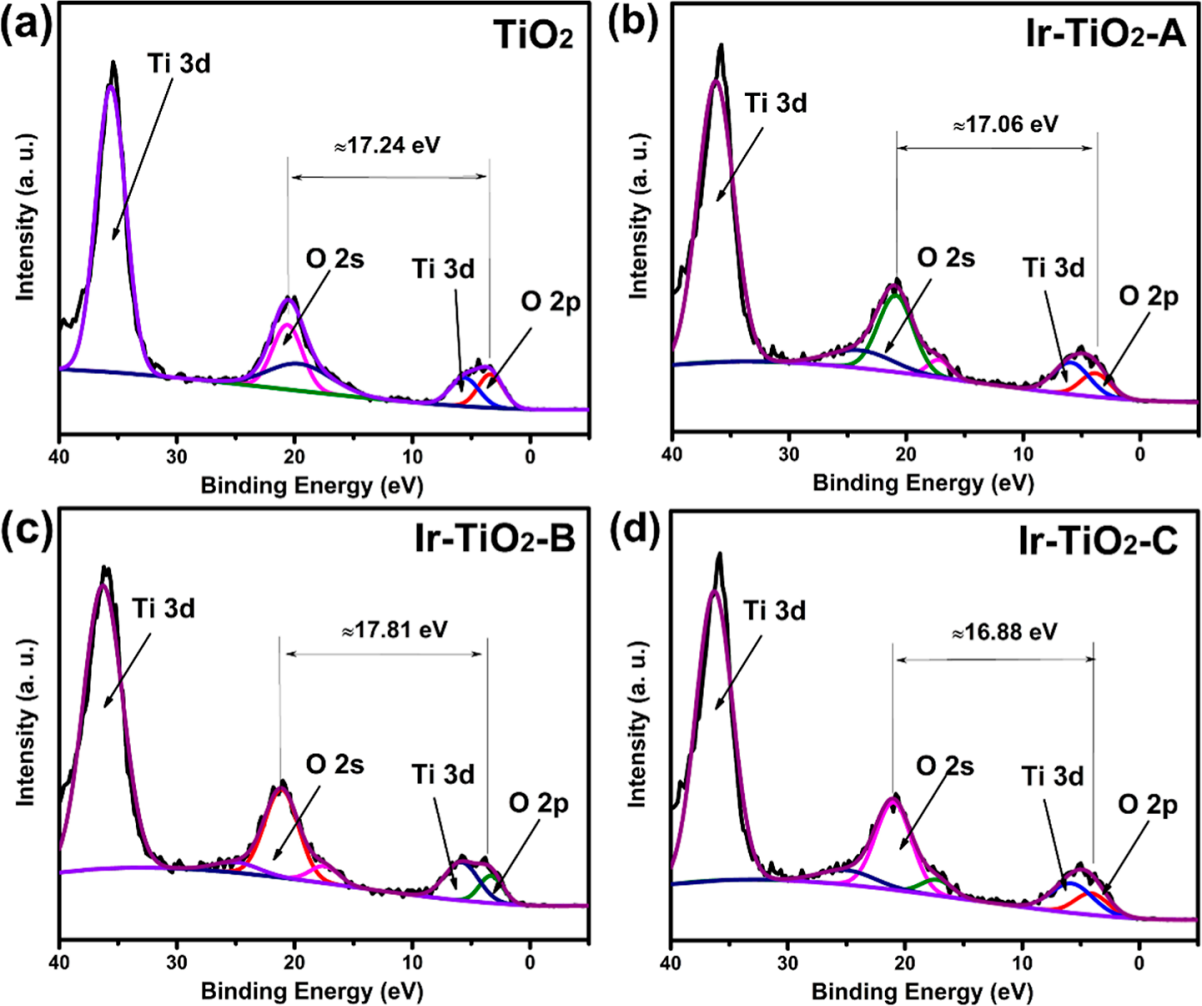
XPS-VB spectra of (a) TiO_2_, (b) Ir–TiO_2_-A, (c) Ir–TiO_2_-B, and (d) Ir–TiO_2_-C.

DFT analysis helped model the band structure and orbital-resolved projected density of states of pristine TiO_2_ and Ir-doped TiO_2_. TiO_2_ has an indirect band gap of magnitude 3.11 eV. Oxygen’s 2p states contribute the most in the valence band maximum, whereas titanium’s 3d states contribute the most in the conduction band minimum. Interesting things happen when TiO_2_ is doped with a strongly correlated transition metal like Ir. Ir has localized d and f orbitals, and its oxide, namely, IrO_2_, is metallic. When Ir replaces Ti as a dopant, energy levels appear near the Fermi level. For sufficiently high doping concentrations as high as 5 at %, Ir-doped TiO_2_ shows metallic nature. At lower Ir concentration, Ti still dominates Ir, Ti 3d hybridizes with Ir 5d, and O 2p interacts with Ir 5p. Figure 4a shows the crystal structure and the orbital-resolved projected density of states of 1% Ir-doped TiO_2_. To achieve such a concentration, a supercell of 5 × 5 × 1 was constructed, resulting in a system with 100 Ti and 200 O atoms. One of the Ti atom was then replaced by Ir. The same convergence criteria as pristine TiO_2_ were used to optimize the structure. The Ir dopant results in deep band gap states, pushing the O 2p states at lower energy than those of TiO_2_.

**Figure 4:**
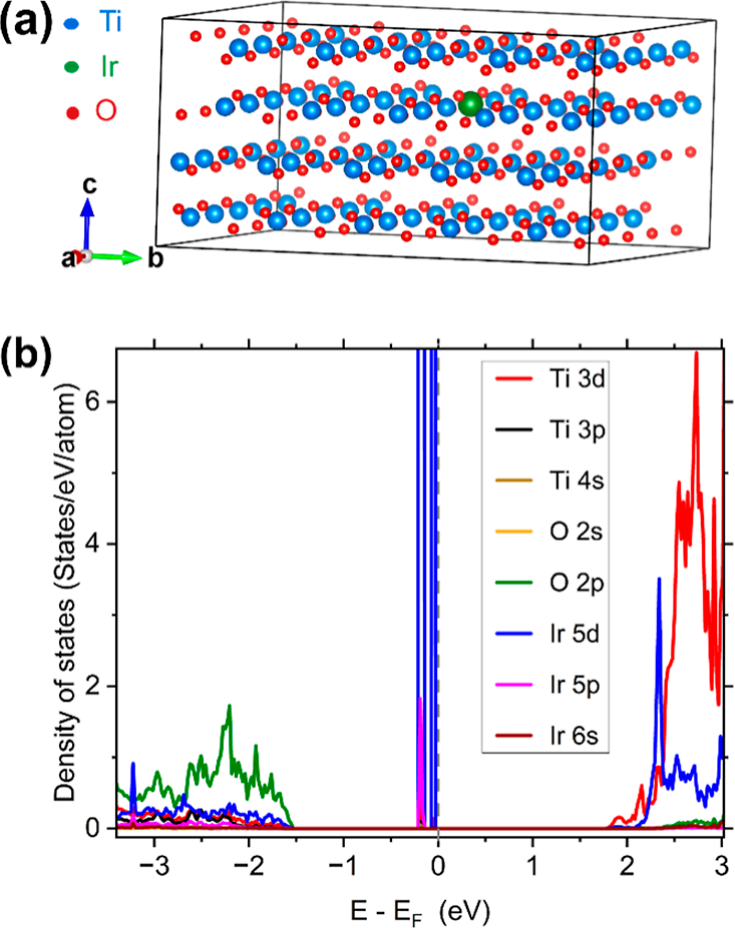
(a) Rotated *a*-axis view of the 1% Ir-doped TiO_2_ unit cell and (b) its orbital-resolved projected density of states.

Due to the importance of light absorption for photocatalytic activity, UV–visible diffuse reflectance analysis revealed an enhancement in light absorption by the synthesized materials. At 400 nm, sample Ir–TiO_2_-C (Figure S7a) exhibited the highest light absorption efficiency compared to the other samples and TiO_2_. All samples demonstrated lower light absorption capability in the UV region compared to TiO_2_, between 200 and about 370 nm. This reflects the ability of iridium to modify the electronic properties of TiO_2_, enhancing its absorption by visible light. As a vital property in photocatalytic materials designed for photocatalysis, the emission property of fabricated materials must be low as a sign of adequate light absorption. This is because high emission of light indicates that photons absorbed from irradiated light are lost due to excited electrons falling back to a lower energy level or the ground state. As expected, our Ir–TiO_2_-C sample (Figure S7b) exhibited a very low photoemission compared with the other material. Ir–TiO_2_-C shows a low electron–hole recombination rate. From diffuse reflectance information, the band gap (Figure S7c) of the material was measured using the Kubelka–Munk function via the improved Tauc plot equation.^36^ The lowering of the band gap from 3.12 eV of TiO_2_ to 2.81, 2.87, and 2.91 eV for Ir–TiO_2_-A, Ir–TiO_2_-B, and Ir–TiO_2_-C, respectively, enables light absorption in the visible range, confirming the effectiveness of the doping. The measured band gap for commercial TiO_2_ is 3.22 eV.

A high yield of hydrogen gas was obtained from the Ir–TiO_2_-C sample with a yield of 1636.7 μmol g^–1^ h^–1^, outperforming all of the other samples (Figure 5a). This yield is about 7 times that of pristine TiO_2_ (238.0 μmol g^–1^ h^–1^), showing the effectiveness of the synthesis material application in hydrogen gas evolution. Samples calcined at 600 °C produced 238.6 μmol g^–1^ h^–1^, 336.6 μmol g^–1^ h^–1^, 709.7 μmol g^–1^ h^–1^, and 48.0 μmol g^–1^ h^–1^ for the Ir–TiO_2_-A, Ir–TiO_2_-B, Ir–TiO_2_-C, and as-prepared samples, respectively. This indicates that the crystalline TiO_2_–amorphous IrO_2_ composite calcined at 500 °C exhibits superior photocatalytic properties compared to the crystalline TiO_2_/crystalline IrO_2_. The high degree of amorphousness in the as-prepared sample resulted in a low yield. The results also confirm the contribution of dopants to modifying the electronic properties of semiconductor materials, thereby enhancing photocatalytic activities. This result is significant compared to a similar study, which yielded 48 to 23.5 μmol h^–1^ g^–1^ of H_2_ evolved.^13^ The advantage of our work can be attributed to the absence of the rutile phase of TiO_2_ and the presence of an amorphous/crystalline pair offering good synergy in terms of charge generation and charge transfer for effective photocatalysis. The presence of anatase and rutile requires the right anatase/rutile phase alignment to form the appropriate heterojunction and internal electric field (IEF) to control charge transfer and recombination.^13,37^ As a result, there must be careful optimization of material fabrication to include Ir, anatase TiO_2_, and rutile TiO_2_ to improve photocatalytic yield, which is less effective in photocatalysis compared to the anatase TiO_2_. Other factors, including good light absorption of our material, favor the broad wavelength of light used in our study. To reduce electron–hole recombination and ensure effective redox activity during hydrogen evolution, methanol (10% v/v) was used as a sacrificial agent. This will help scavenge holes (h^+^) generated at the VB, ensuring that more free electrons (e^–^) are available for reducing protons at the CB for producing H_2_ from water.

**Figure 5:**
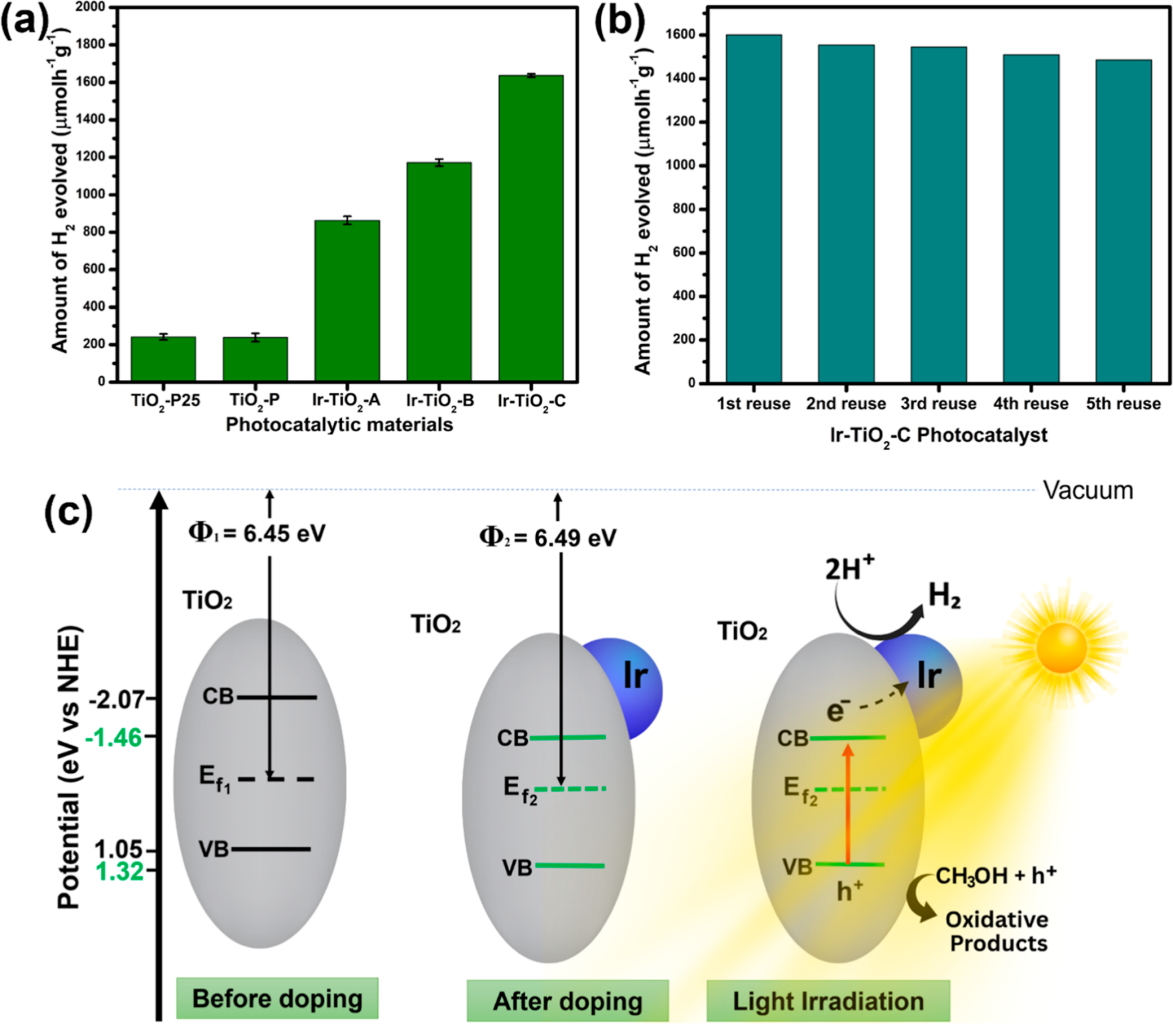
(a) Evaluation of photocatalytic hydrogen evaluation results for samples, (b) stability results of the Ir–TiO_2_-C sample after 5 cycles, and (c) schematic illustration of the mechanism of photocatalytic hydrogen evolution using Ir–TiO_2_ composite nanomaterial.

Figure 5c shows a schematic representation of the mechanism of charge carrier generation, separation, migration, and active site photocatalytic reaction for the Ir–TiO_2_-doped photocatalyst. The Ir serves as a photoexcited electron-capturing site, which lowers electron–hole recombination. The mechanism of the crystalline TiO_2_–amorphous IrO_2_ composite will result in the formation of an internal electric field (IEF) due to the difference in the work function. The work function of the amorphous IrO_2_ is expected to be about 4.23 eV,^23^ as previously reported, which is far lower than TiO_2_ (6.45 eV), as experimentally determined. This will cause electrons to flow from the amorphous IrO_2_ to TiO_2_ when combined. There will be continuous electrons flowing to the TiO_2_ until their Fermi energy levels reach equilibrium,^38,39^ where an electron depletion layer and an electron accumulation layer will form near the interface between the IrO_2_ and TiO_2_, respectively, causing the IrO_2_ to be positively charged and the TiO_2_ to be negatively charged. An IEF is formed and directed from IrO_2_ to TiO_2_. During visible light irradiation, electrons are photoexcited from the valence band (VB) to the conduction band (CB). The creation of IEF will strongly prevent the recombination of electrons^40^ photogenerated from the CB of the IrO_2_ to the CB of the TiO_2_ after light irradiation. Due to this phenomenon, photogenerated charge carriers will be retained in the amorphous IrO_2_/crystalline TiO_2_ composite to participate in photocatalytic water splitting to generate hydrogen gas.

After the hydrogen experiment, the samples were observed to be highly recoverable via washing with distilled water, followed by ethanol, and then drying in an oven at 60 °C, allowing for reuse. Investigation into the stability of the material revealed a high performance after five cycles (Figure 5b). The excellent recyclability and reusability of our materials demonstrate their long-term application, efficiency, and economic importance, particularly in cost-saving applications arising from the high cost of iridium precursors. Characterization results after the fifth cycle (Figures S8–S10) show no significant difference in the sample, indicating that the physicochemical properties of the photocatalyst are retained.

## 4 Conclusions

In this work, we successfully fabricated anatase TiO_2_ loaded with iridium using a simple yet highly effective one-pot solvothermal synthesis. Varying the amount of iridium component significantly contributed to identifying different phases of our materials, primarily iridium-doped titania (Ir–TiO_2_) and an amorphous IrO_2_/crystalline TiO_2_ composite. The possible formation of residual carbonaceous species from the decomposition of the F127 surfactant suppressed the crystallinity of IrO_2_, causing it to remain amorphous at 500 °C. The TiO_2_ remained highly crystalline; therefore, the effect can be attributed partly to the low loading of the Ir component in the composite and the possible presence of residual carbonaceous species. Characterization techniques such as SEM, TEM, and BET assisted in unveiling the morphology, particle size, and porosity of the formed materials. Electrochemical characterization assisted in understanding the electronic properties of the samples. This study revealed the role of iridium in modifying the electronic properties of TiO_2_, thereby reducing the band gap of TiO_2_ and enhancing its light absorption, stability, and photocatalytic performance. DFT simulation revealed oxygen’s 2p states contributing the most in the valence band maximum, whereas titanium’s 3d states contribute the most in the conduction band minimum. Incorporating iridium as a dopant replaces titanium, introducing energy levels near the Fermi level, which contributes to narrowing the band gap and modifying the chemical and electronic properties of TiO_2_. Results from the solar light water splitting experiment for hydrogen evolution produced 1636.7 μmol h^–1^ g^–1^ over 3 h of activity for the best sample (Ir–TiO_2_-C). This yield is almost 7 times that of pristine TiO_2_ (238.0 μmol h^–1^ g^–1^). Based on the findings, it can be concluded that Ir significantly improved the photocatalytic performance of TiO_2_. The high material stability, recyclability, and reusability of our material also make it highly economical for use in hydrogen evolution regardless of the cost of the iridium precursors.

## Supplementary Material



## Data Availability

Data will be made available upon request.

## Notes

Additional information on FESEM images, FESEM–EDX images, XRD spectrum, FTIR spectrum, UV–vis DRS absorption and reflectance spectra, PL spectrum, TGA plot, XPS spectra, BET analysis results, and characterization results after catalyst reuse (PDF)
