# Supplementary material for: Harnessing Visible Light: Unraveling the Photocatalytic Water Splitting Activity of Ir–TiO2
Source: ACS Appl Energy Mater. 2025 Aug 26;8(17):12733–40. doi: 10.1021/acsaem.5c01776 (PMC12421512; doi:10.1021/acsaem.5c01776)
Supplement: Supplementary file 1 [file ae5c01776_si_001.pdf]

Supporting Information

Harnessing Visible Light: Unraveling the Photocatalytic Water Splitting Activity of Ir-TiO_2

Moses D. Ashie[1], Chandra M. Adhikari[2], Gayani Pathiraja[3] and Bishnu Prasad Bastakoti[1]*

[1]Department of Chemistry, North Carolina A&T State University, Greensboro, NC 27411, USA
[2]Department of Chemistry, Physics, and Materials Science, Fayetteville State University, Fayetteville, NC 28301, USA
[3]Department of Nanoscience, Joint School of Nanoscience and Nanoengineering, University of North Carolina at Greensboro, 2907 East Gate City Blvd, Greensboro, NC 27401, USA

Email: bpbastakoti@ncat.edu

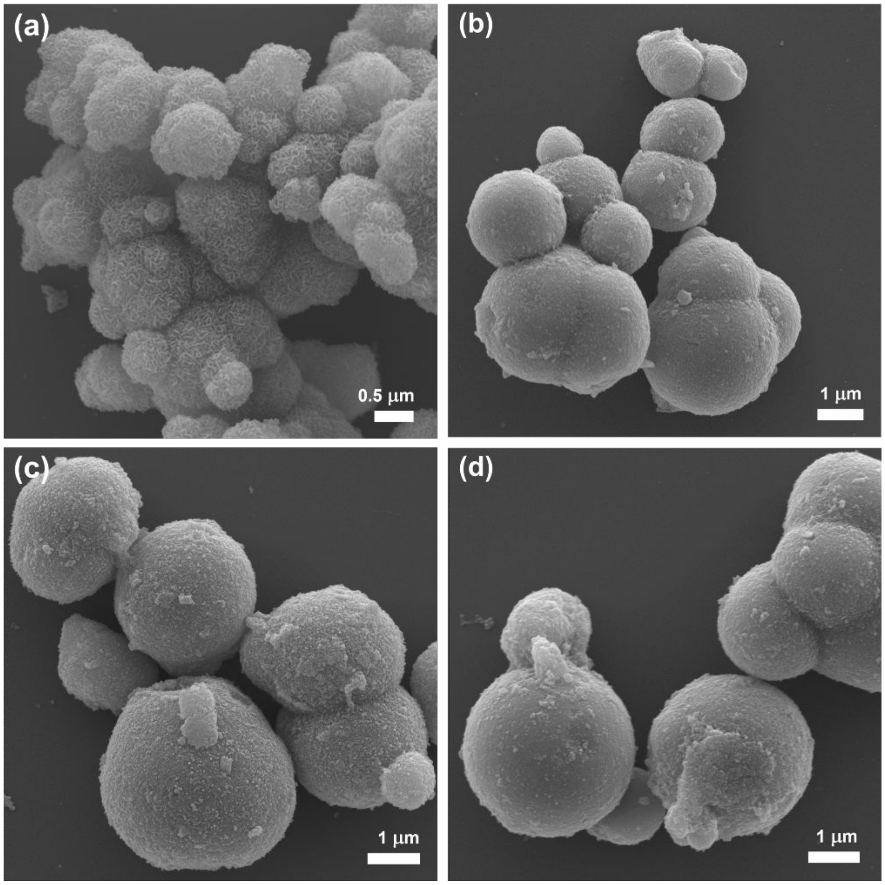

Figure S1: SEM of a) TiO_2, b) Ir-TiO_2-A, c) Ir-TiO_2-B, and d) Ir-TiO_2-C samples synthesized using F-127.

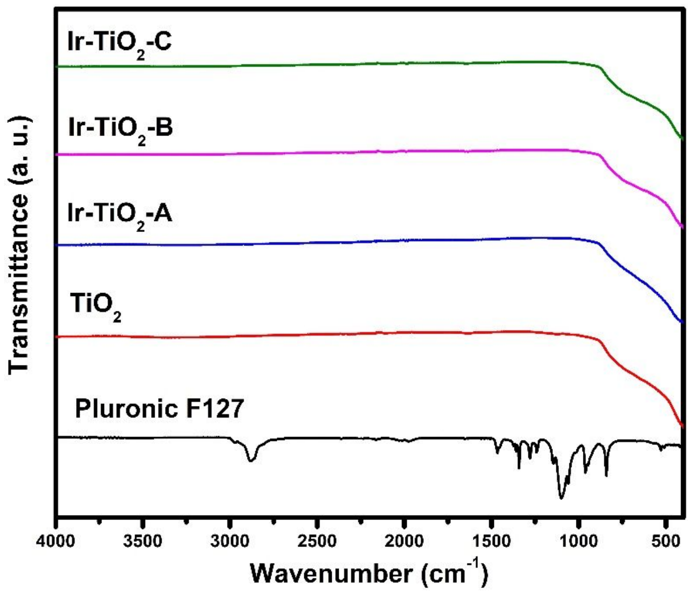

Figure S2: FTIR of Pluronic F127, and calcined TiO_2 and Ir-TiO_2 samples.

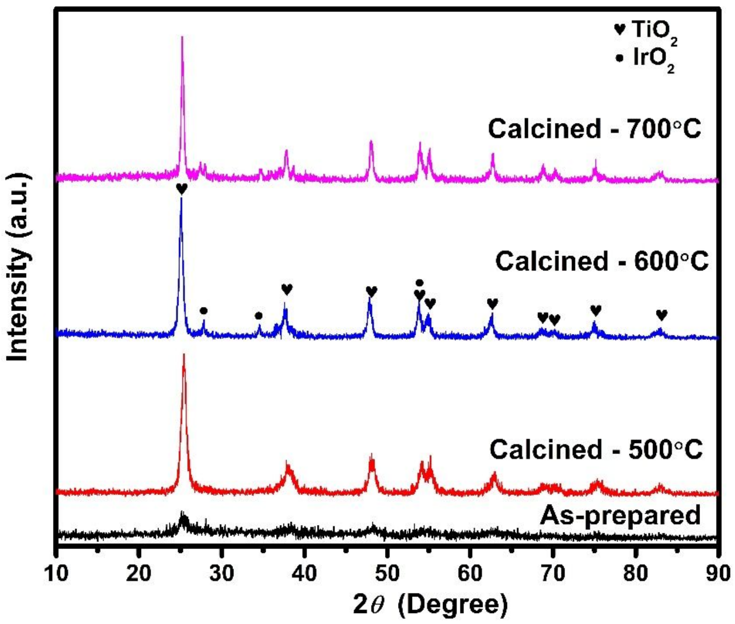

Figure S3: XRD of as-prepared Ir-TiO_2-C sample and Ir-TiO_2-C samples calcined at different temperatures.

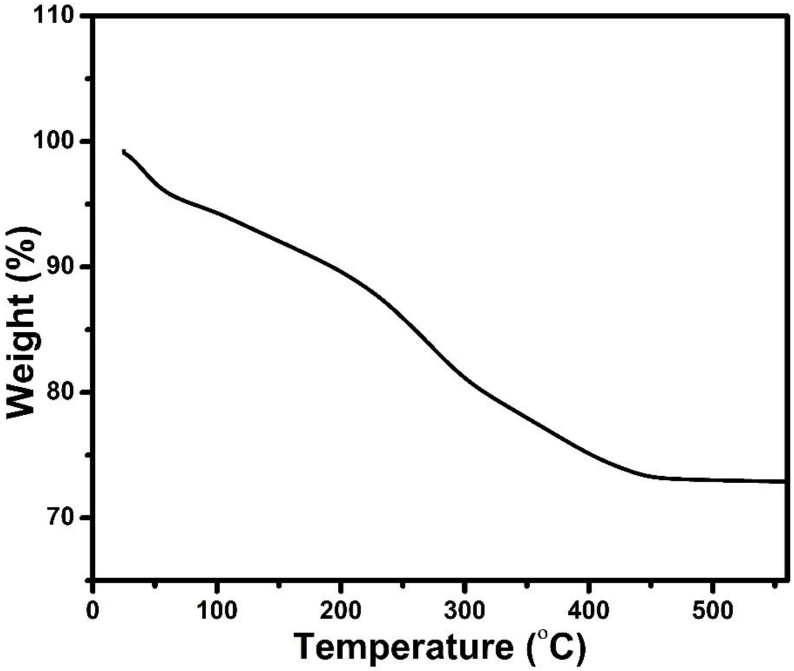

Figure S4: TGA plot of surfactant-assisted Ir-TiO_2-C sample.

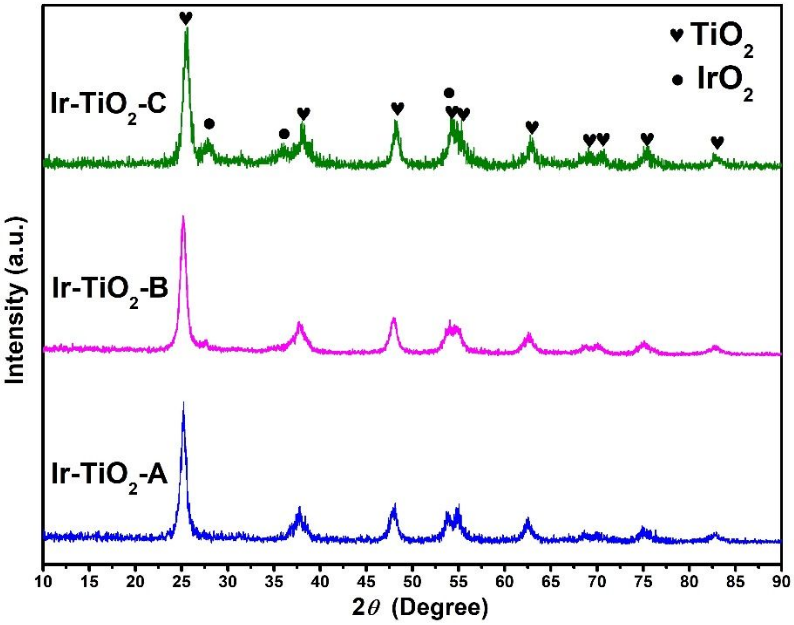

Figure S5: XRD of Ir-TiO_2 samples calcined at 500 °C. The samples were synthesized in the absence of F127 surfactant.

Table S1. Summary of BET surface area and pore sizes of TiO_2-P, Ir-TiO_2-A, Ir-TiO_2-B, and Ir-TiO_2-C composite samples.

**Sample**	**BET Surface area / m^2g^{-1}**	**BJH Pore size (Adsorption) / nm**
TiO_2-P	106.9	9
Ir-TiO_2-A	69.9	6.2
Ir-TiO_2-B	82.3	6.4
Ir-TiO_2-C	112	6.8

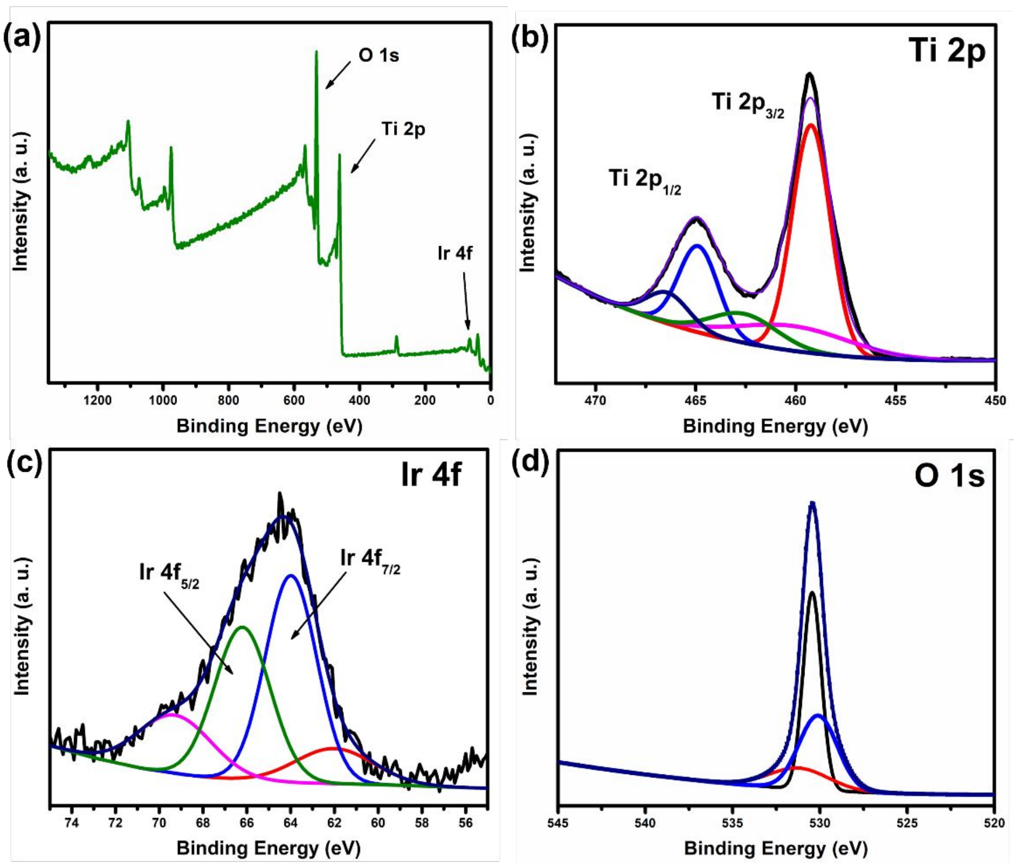

Figure S6: High-resolution XPS results of Ir-TiO_2 a) Survey b) Ti 2p, c) Ir 4f, and d) O 1s.

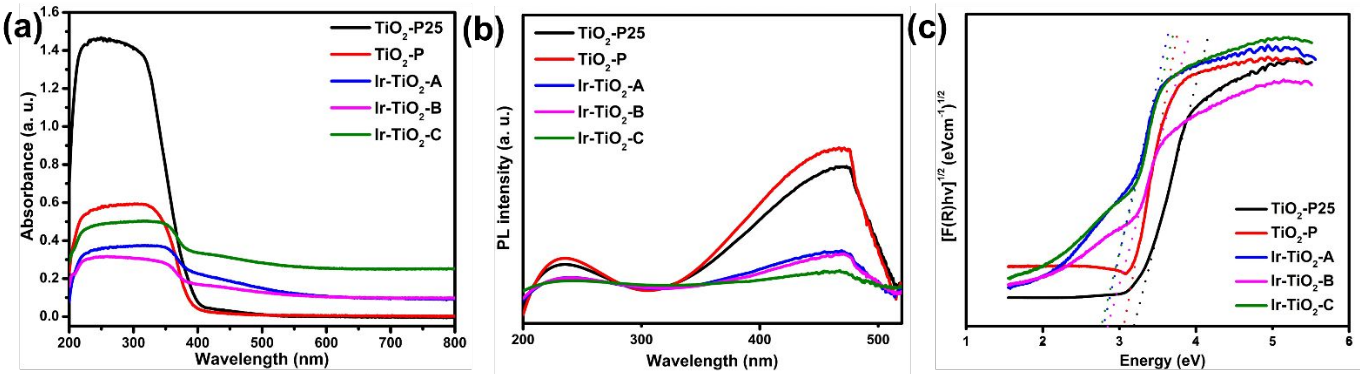

Figure S7: a) UV–Visible spectroscopic absorption spectra, b) photoemission spectra, and b) band gap determination of TiO_2-P25, TiO_2-P, doped Ir-TiO_2-A, Ir-TiO_2-B, and Ir-TiO_2-C composite materials.

<u>Sample recovery after hydrogen evolution experiment</u>

After each analysis, the samples were centrifuged at 7500 rpm for 5 min. The collected samples were washed and dried in an oven at 60 °C for 12 h.

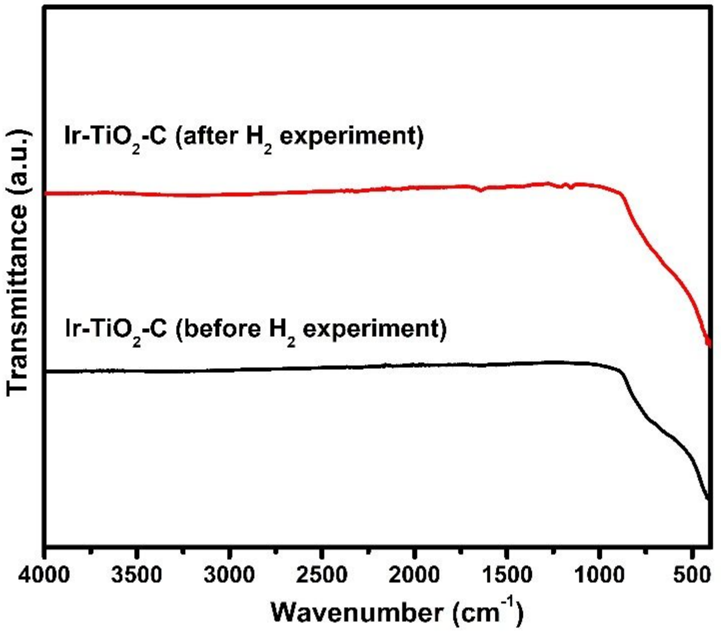

Figure S8: FTIR spectrum of Ir-TiO_2-C sample after multiple re-use.
The FTIR peaks observed at about 1153 cm^{-1}, 1215 cm^{-1}, and 1640 cm^{-1} are characteristic of C-O and C=O bond stretching vibrations (1) respectively. The low-intensity peaks also indicate a lower amount of these surface adsorptions. Ref: (1) Instanano. FTIR Functional Group Database Table. 2025;2025. Available from: https://instanano.com/all/characterization/ftir/ftir-functional-group-search/

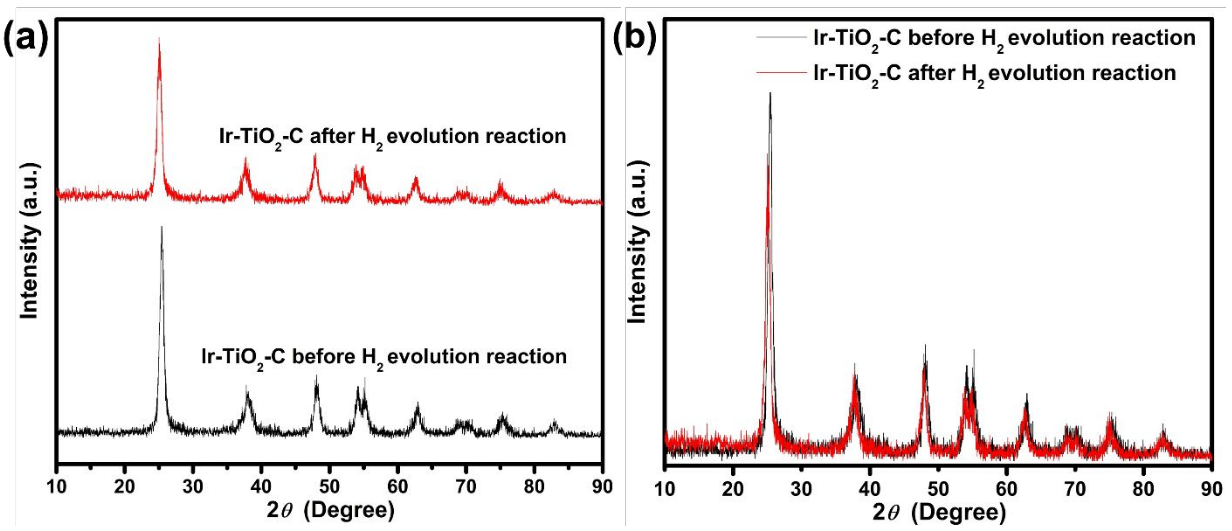

Figure S9: Stability of Ir-TiO_2-C sample after all cycles of hydrogen evolution activity. The XRD pattern before and after hydrogen evolution experiments shows a negligible difference, with only a slight shift of all peaks towards a lower angle. This can be due to interactions with traces of molecules from the sacrificial agent.

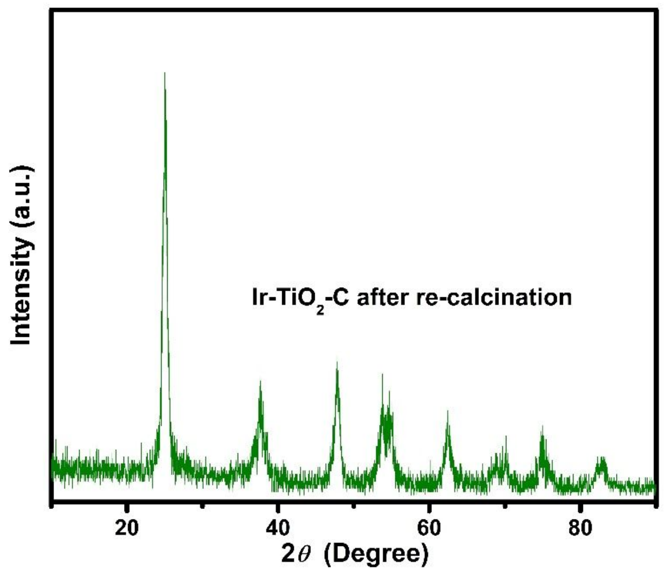

Figure S10: XRD spectrum of re-calcined Ir-TiO_2-C sample after re-cycling.
